# Behavioural Profiles in Captive-Bred Cynomolgus Macaques: Towards Monkey Models of Mental Disorders?

**DOI:** 10.1371/journal.pone.0062141

**Published:** 2013-04-29

**Authors:** Sandrine M. J. Camus, Catherine Blois-Heulin, Qin Li, Martine Hausberger, Erwan Bezard

**Affiliations:** 1 Université de Bordeaux, Institut des Maladies Neurodégénératives, UMR 5293, Bordeaux, France; 2 CNRS, UMR 6552 Ethologie Animale et Humaine, Université de Rennes 1, Paimpont, France; 3 Institute of Lab Animal Sciences, China Academy of Medical Sciences, Beijing, China; 4 Motac Neuroscience Ltd, Manchester, United Kingdom; 5 CNRS, UMR 6552 Ethologie Animale et Humaine, Université de Rennes 1, Rennes, France; 6 CNRS, Institut des Maladies Neurodégénératives, UMR 5293, Bordeaux, France; INSERM/CNRS, France

## Abstract

**Background:**

To date, experimental and preclinical studies on neuropsychiatric conditions have almost exclusively been performed in experimentally-induced animal models and have only rarely relied upon an ethological approach where animals have been observed in more naturalistic settings. The laboratory species of choice has been the rodent while the potential of more closely-related non-human primates have remained largely underexplored.

**Methods:**

The present study, therefore, aimed at investigating the possible existence of spontaneous atypical/abnormal behaviours displayed by 40 cynomolgus macaques in captive conditions using an unbiased ethological scan-sampling analysis followed by multifactorial correspondence analysis and a hierarchical clustering.

**Results:**

The study identified five distinct profiles (groups A to E) that significantly differed on several behaviours, body postures, body orientations, gaze directions and locations in the cage environment. We suggest that animals from the low n groups (D and E) present depressive-like and anxious-like symptoms, reminiscent of depressive and generalized anxiety disorders. Inter-individual differences were highlighted through unbiased ethological observations of spontaneous behaviours and associated parameters, although these were not associated with differences in plasma or cerebrospinal fluid levels of either stress-related hormones or monoamines, i.e. in accordance with the human situation.

**Conclusions:**

No interventional behavioural testing was required to discriminate between 3 typical and 2 atypical ethologically-defined behavioural profiles, reminiscent of certain depressive-like and anxiety-like symptoms. The use of unbiased behavioural observations might, thus, allow the identification of animal models of human mental/behavioural disorders and their most appropriate control groups.

## Introduction

The past twenty years have seen unprecedented efforts to investigate the pathophysiology of mental disorders. Despite huge progress in our understanding of the neurobiological and molecular substrates of these disease states, there are significant difficulties in translating this basic science knowledge into therapeutic advances [1]. Although not specific to biological psychiatry, this stems from the etiologic complexity of mental disorders. Understanding the specific involvement of the environmental, genetic and other individual susceptibility factors in their development [2]–[5] requires factor-controlled experiments in animal models.

To date, experimental and preclinical studies have almost exclusively been performed in rodents using “induced” (genetic e.g. [6], lesional e.g. [7], or pharmacological e.g. [8] manipulations) rather than “spontaneous” paradigms (ethological models living in naturalistic/experimental settings e.g. [9]). Although the usefulness of these models is undeniable, the etiologic processes and the resulting neural and behavioural features meant to replicate those of human diseases are restricted, thereby limiting the construct validity of those models [1]. Further difficulties emerge from the methodological gap between preclinical and clinical fields. The current Diagnostic and Statistical Manual of Mental Disorders (DSM IV) [10] classifies diseases based upon a wealth of symptoms verbally reported by patients or their relatives but not directly observed in patients’ daily life environment. Conversely, only observable behaviours and associated features can be assessed in animal models. “Symptomatic” correlates between humans and animals are thus more than arguable. Anatomical, biochemical, neuropathological or behavioural similarities between human diseases and animal models, namely the face validity of a model, are, therefore hardly achievable [1], [5] without a translational element that can confirm their relevance.

These considerations led us toward more “spontaneous” occurrence of pathological behavioural profiles in animal populations. Most farm animal species live in suboptimal conditions and express their lack of well-being either by changing the frequencies of usual behaviours or by developing unusual activities e.g.[11]–[13], such as stereotypic behaviours (SB) defined as “repetitive and invariant behaviours with no apparent goal or function” [14]. Captive carnivores in small zoo enclosures express more motor SB (pacing, circling or head-tossing) than those in large enclosures [15], [16]. Pet animals such as gerbils display stereotypic digging when housed in cages devoid of shelters [17]. The vast majority of macaques used either in toxicology (95% of total use in research) or in basic science research (5%) in Japan, USA and Europe originate from Asian breeding facilities (p29 and p114 in [18]). These breeding farms might be considered as a naturalistic environment to long-tailed macaques regarding several wild-like features, such as mixed-gender mixed-generation social groups with a natural formation of stable ranking hierarchies and the seasonal birth of several infants. Other parameters are however at odds with the ecology of the species in the wild. Key differences of relevance to our current topic are an early weaning of the young (5–6 months old), their following peer-rearing and a pre-shipment single housing for a variable period of time. These gregarious animals indeed normally display highly heritable matrilineal hierarchies based upon social transmission and signals that need to be displayed at appropriate times [19]–[22]. These standard and worldwide accepted procedures are likely stressful and provide an environment to study “spontaneous” (in opposition to the induced models reported in the previous paragraph) inter-individual differences in the expression of atypical behavioural profiles among a huge number of individuals, without actually having to interfere with their usual housing conditions, thereby mimicking the adverse life events likely involved in the development of depressive disorders in Humans [3], [23]. Captivity by itself is a manipulation of the environment, since it necessarily induces behavioural modifications (e.g. decrease of foraging induced by regular feeding schedule, or decrease of locomotion induced by limited space and the impossibility to search for a nesting spot) compared to wild animals [24]. However it is required in any animal models in order to control the largest amount of environmental parameters. Here we introduced the “spontaneous” aspect of our study in opposition to animal models induced with invasive methods requiring direct manipulations of the individuals (e.g. genetic modifications, cerebral lesions or chronic administration of molecules). These latter models also imply captive housing conditions with fixed feeding schedule and reduced living areas compared to the animal wild territories (and likely similar housing history if the animals provide from breeding farms), thereby allowing us to consider the effects of husbandry as equivalent in both models. The authors remain however aware that “spontaneous” refers to the lack of human interventions apart from the common husbandry processes, inevitable in every experimental setting.

Since Harlow’s crucial experiments on the effects of early social stress in the 1960’s [25]–[27], there have been few studies into how non-human primates (NHPs) express an altered well-being in terms of behavioural repertoire, atypical behaviours, and SB in naturalistic paradigms. Shively et al have extensively studied a model of social stress-associated depression in cynomolgus monkeys, consuming a saturated fat- and cholesterol-enriched diet and submitted to a manipulation of social status, where increased levels of immobility occur as well as a slumped body posture in which monkeys appear to be withdrawn from the environment [28]–[30]. The variable foraging demand paradigm (i.e. macaque females are submitted to unpredictable food availability and have to intensively investigate a complex apparatus to find food, thereby disrupting mother-infant interactions) has also been often used to study depressive-like and anxiety-like behaviours in the offspring, which expressed less play and investigation behaviours and seek contact with their mother more often than normally-reared infant [31], [32]. Being phylogenetically, anatomically and behaviourally close to humans, NHPs are the most appropriate choice to mimic complex mental disorders [33]–[37]. However, while widely acknowledged as the ideal model for the study of neurological disorders [38], “relative to both rodent-based models and direct human studies, psychiatric research using NHPs remains comparatively underrepresented in the literature” [34]. Possible reasons for this are that, not only is an unbiased methodology for the identification of such spontaneously occurring disorders in breeding facilities lacking, but also researchers would require access to large numbers of NHPs accommodated in standardized conditions.

This preliminary study availed of these breeding farms and, therefore, aimed at investigating the existence of atypical behaviours, possibly reminiscent of human mental disorders, displayed by cynomolgus macaques housed in farming conditions using an unbiased ethological analysis. Such conditions, employed by breeding and supply establishments, included single housing and timed food provisioning. We hypothesized that in these farming conditions, individual differences would emerge that may mimic human diversity of responses to stressful situations.

## Materials and Methods

### Animals and Housing Conditions

Forty 3-year-old male cynomolgus monkeys (Macaca fascicularis) were studied in a breeding farm (Hannan Jingang Laboratory Animal Corporation, Hannan Province, People’s Republic of China).

Prior to the study, the breeding processes involved a social one-male, multi-female grouping. The young were weaned around 6-months old and then group-housed with peers until 3 years of age. After this time, the males were singly-housed in cages of dimensions L70 cm x W60 cm x H80 cm, with visual, auditory and olfactory contacts with their peers. Manual contacts with the adjacent neighbors were logically impossible because of the opaque side walls of the cage. However some interactions occurred through the top of the cage or when monkeys shifted their wheeled cage a few centimeters. They had been singly-housed for 9 months prior to the beginning of the study. In such breeding farms, animals are housed in single cages before being dispatched to third party users, mostly the toxicology industry (>90%) and the experimental research. Animals were fed monkey pellets three times a day and fruit once daily. The water trays were filled at each feeding time. The animals were reared indoors but with natural lighting.

### Ethics Statement

The institutional animal care and use committee of the Institute of Lab Animal Science of Chinese Academy of Medical Science approved this study. The housing conditions were in compliance with the guidelines of the Haikou Forestry Office (Hannan Province, People’s Republic of China). Such conditions correspond to standard practices in operation in breeding facilities providing macaques to the whole Japanese, American and European toxicology industry and research laboratories. Veterinarians skilled in the healthcare and maintenance of non-human primates supervised animal care. No animal was harmed or killed in the course of the experiments.

### Behavioural Assessment

Macaque behaviour was video-recorded and observed outside the feeding and cleaning times, in a randomized order at three time points (morning, noon and afternoon), on two non-consecutive days (6 sessions per individual). We used a scan-sampling method, appropriate for time budgeting [39], in which behavioural parameters were assessed every 2 minutes during 30-minute sessions, resulting in 90 scans per individual. A unique trained observer (SC; intra-observer reliability: Spearman rank order correlation R = 0.987) spent 12 hours in each observation room to collect the data. She was facing the door or window at all time rather than the cages and looked at the camera screen rather than at the individuals (a gaze directed to a macaque’s eyes being interpreted as a threat). We focused on behavioural profiles rather than single items. Inspired from 2 published studies [24], [40] and completed with any additional items observed during our observations, two repertoires were constructed: one reporting the interaction with the environment (**Table 1**) and one describing the position within the environment (**Table 2**). We investigated the percentages of occurrence of each item with regard to the 90 scans in order to obtain mean behavioural and postural time budgets, body orientation and location profiles. We also reported the behavioural diversity (the number of different behaviours expressed during the 90 scans) and the percentages of behavioural switch between successive scans (each scan was scored: 0 if the behaviour was the same as in the previous scan, or 1 if it was different; the scores were added up within one session and transformed in a percentage with regard to the 15 scans of a session).

**Table 1:** Single-housed cynomolgus monkey behavioural repertoire and time budget.

Grouped behaviours for MCA	Detailed collected behaviours	%±SEM
**inactivity**	is not engaged in any other behaviours, with open eyes	**30.9±3.3**
	rests: not engaged in any other behaviours, with closing or closed eyes	
	tensed inactivity (usually after an aggressive encounter)	
**investigation/manipulation**	manipulates collar non-repetitively with hands and/or mouth	**17.5±1.9**
	manipulates feeding tray with hands and/or mouth non-repetitively	
	uses feeding tray as a mirror, looks into it	
	manipulates other object with hands and/or mouth non-repetitively	
	investigates cage (searches, sniffs wall or bars)	
**maintenance behaviours**	maintenance behaviours (urinate, defecate, rub eyes)	**8.4±1.0**
	bites its nails	
	rubs hands on bars or floor	
	selfgrooms (grooming of own body)	
**locomotion**	Locomotion: change of location without any other behaviours	**5.4±1.1**
**social behaviours**	grooms neighbour (allogrooming) - affiliative	**3.9±0.6**
	is groomed by neighbour (allogrooming) - affiliative	
	presents genitals in a non-sexual context - affiliative	
	other affiliative behaviours (facial expression, seak positive contact with peer)	
	threatens or hits peer - aggressive	
	displays submissive facial expression - submissive	
**behaviours toward human**	interacts with observer (threat, submission, lipsmacking, genital display)	**3.4±1.1**
**displacement behaviours**	vacuum chews (chews despite empty mouth and cheekpouches)	**9.3±1.0**
	yawns	
	scratches self	
**cage shake**	shakes own or neighbour’s cage	**5.6±1.1**
**stereotypic behaviours**	manipulates collar repetitively	**15.0±2.1**
	manipulates feeding tray repetitively	
	manipulates other object repetitively	
	gnaws bars repetitively	
	licks bars repetitively	
	licks own tail or other body part repetitively	
	bites own tail repetitively	
	motor stereotypy (pacing, flipping…)	
	swings from left to right repetitively	
	moves head up and down repetitively	
	moves head repetitively in another way	
	moves repetitively from biped to crocodile postures	
	clings on genitals repetitively	
	oral stereotypy (tongue movement or tongue chew)	
	self suckling	
**feeding behaviours**	“hunts” insect (try or manage to catch)	**4.7±0.8**
	eats or drinks	
**sexual behaviours**	typical sexual behaviour (masturbate)	**0.5±0.2**
	atypical sexual behaviour (masturbate neighbour)	

Collected detailed items (adapted from [24], [40]) were then grouped for multiple component analysis (MCA). Considering the 40 individuals, the mean percentages of occurrence and standard error means (SEM) were calculated for each grouped behaviour and represented the time budget of this single-housed population (right column).

**Table 2:** Location, gaze direction, body posture and orientation items displayed by single-housed cynomolgus monkeys.

Variables for MCA	Detailed collected variables	% ± SEM
**Gazes**		
**observer**	observer	**28.6±2.3**
**still environment**	wall	**19.4±2.2**
	ground or ceiling	
**object/self**	manipulable object (feeding or water tray, cage lock)	**16.2±1.5**
	own body	
**living environment**	peer	**35.7±2.2**
	outside	
	insect	
**Locations in cage**		
**front**	front or back (cage depth divided in 2 virtual parts)	**51.7±4.5**
**back**		**48.3±4.5**
**up**	up or bottom (cage height divided in 2 virtual parts)	**25.6±2.9**
**bottom**		**74.4±2.9**
**sides**	sides or middle (cage width divided in 3 virtual parts)	**68.2±3.0**
**middle**		**31.8±3.0**
**Postures**		
**biped**	standing on hind limbs (biped)	**4.5±0.8**
**seated**	resting on the buttocks with straight back	**61.2±2.8**
	resting on the buttocks with bent back	
	resting on the buttocks with stretched legs	
**on bars**	seated posture but on bars	**22.0±2.9**
	upside down four-legged (hanging on ceiling bars)	
	suspending in any other way (four limbs on bars)	
**four-legged**	four-legged, hanging tail	**6.6±1.0**
	four-legged, tail in “?” shape	
	four-legged, tail above head	
	four-legged, straight tail (in the continuity of the back)	
	« bottom up » (four-legged with head on the ground level)	
**slumped**	slumped (seated head lower than shoulder's line)	**4.6±0.9**
**crouched**	crouched (ventral surface close to floor; head at or below the level of the shoulders)	**1.1±0.2**
**Body orientations**		
**outside**	exterior/observer	**59.5±2.8**
**ground**	ground or ceiling	**9.1±1.3**
**wall**	wall	**31.3±3.0**

Collected detailed items were then grouped for multiple component analysis (MCA). Considering the 40 individuals, the mean percentages of occurrence and standard error means (SEM) were calculated for each grouped variable (right column).

### Factor Analyses

As behavioural data were not normally distributed, they were submitted to multifactorial correspondence analysis (MCA see **Figure S1**; SPAD© 7.4, Coheris) that uses chi-square criterion to assess differences and similarities between frequencies of qualitative variables. Active variables are placed in a multidimensional cloud in which two items are at a short distance if they show similar proportions in the same individuals and conversely they are distant if expressed by different individuals. The same process is then repeated with individuals. Two individuals are close if they share similar behavioural profiles. Both clouds are then displayed together by projection onto planes, defined by factors. Each factor accounts for a certain proportion of the total variance of the cloud [16]. We here used grouped behaviours, grouped body postures, body orientations and locations as active variables (**Tables 1** **and** **2**). A hierarchical clustering analysis was then performed on the coordinates in the individuals’ cloud to describe inter-individual similarities [41]. This analysis sorts individuals on the dimensions defined by the previous MCA and creates clusters that maximize within-group similarity and minimize between-group similarity [41]. For each resulting cluster of individuals, the mean occurrence percentage of each behavioural item was calculated and reported on radar graphs.

### Physiological Sample Collection and Assay

Using restraint, blood and CSF samples were collected between 9 and 11 am, at least 48 h after the last observational session. No anesthetic was administered prior to sampling to avoid any chemical interference with the measured molecules. Blood was obtained via saphenous venipuncture, collected in EDTA tubes (5 mL) and centrifuged at 4600 rpm for 10 min at 4°C. Plasma was then stored in an Eppendorf tube. CSF was collected into Eppendorf tubes (1 mL) by inserting a 22 gauge spinal needle above the terminal phylum following published protocols [42], [43], and centrifuged at 3200 rpm for 10 min at 4°C. Plasma and clean CSF were stored at -80°C until assayed. Plasmatic levels of cortisol and adrenocorticotropic hormone (ACTH) were determined by enzyme immunoassay (Cortisol EIA kit, Enzo Life Sciences) and fluorescent-coded magnetic bead immunoassay (Milliplex MAP Non-Human Primate Pituitary Magnetic Bead Panel, Millipore) respectively. For both measurements, all samples were run in duplicate according to the manufacturer’s guidelines. CSF samples were assayed by high performance liquid chromatography following published protocols [44], [45] to measure the levels of monoamines and their metabolites: serotonin (5 HT) and 5-hydroxyindoleacetic acid (5-HIAA), dopamine (DA) and homovanillic and 3,4-Dihydroxyphenylacetic acids (HVA and DOPAC), and norepinephrin (NE). The mean physiological concentrations were calculated for each behaviourally-discriminated group.

### Statistical Analyses

The statistical analyses of the collected data were conducted using Statistica^©^ 8.0 (StatSoft, Inc.). As data were not normally distributed, non-parametric Kruskal-Wallis ANOVA (Table S1) and Mann-Whitney U tests were used to compare behavioural and physiological variables between clusters of individuals. Multiple tests were performed, a Bonferroni adjustment was thus applied to keep the type I error constant. The accepted P level becomes the α probability divided by the number of hypothesis tests: 0.005 (when 10 hypotheses). Considering the risk of masking significant effects following the correction because of the low n and limit of validity for corrections below n = 5, we chose to report also the cases of approximations to statistical significance (P<0.05). A correction for small group size was also applied when the group contained less than 10 individuals.

## Results

Behaviours, gaze directions, locations in the cage, body postures and orientations were collected from 40 singly-housed macaques using a scan sampling method. The mean behavioural time budget of the monkeys (**Tables 1** and **2**) is indicative of the main behaviours expressed in such housing conditions: 30.9% (±3.3%) of time spent inactive, 17.5% (±1.9%) investigating the cage, 15.0% (±2.1%) in stereotypic behaviours (SB) and 8.4% (±1.0%) in maintenance activities. The main displayed postures were seated (61.2±2.8%) and “on bars” (22.0±2.9%). The animals were oriented towards the observer in 59.5% (±2.8%) of the scans and mainly located in the bottom side parts, either at the front or the back of the cage (sides: 68.2±3.0%, bottom: 74.4±2.9%, front: 51.7±4.5%).

**Figure 1:**
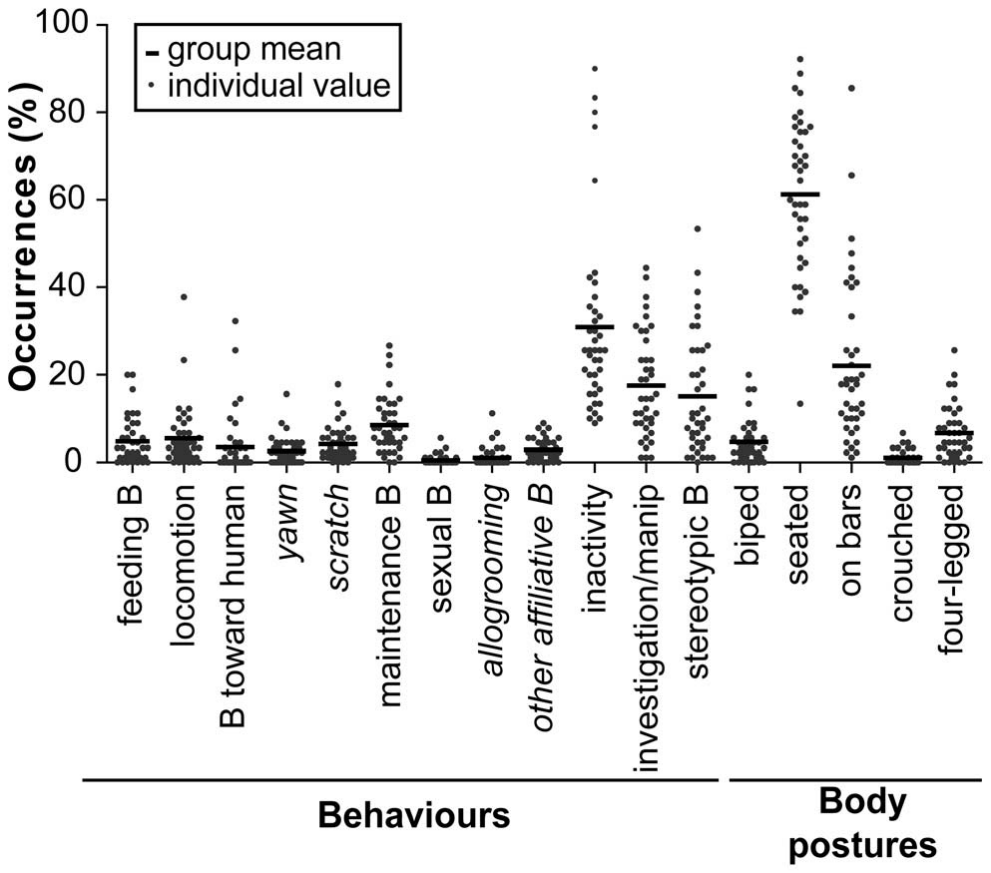
Behavioural and postural time budgets of single-housed cynomolgus monkeys. The percentages of occurrence with regards to the total number of scans were calculated for each collected variable. A few collected (in *italics*) or grouped (regular font) variables are reported in this graph. Grey spots indicate individual values while black lines indicate the mean of the 40 individuals. “B” and “manip” stand for “behaviour” and “manipulation”. See **Tables** **1** **and** **2** for a detailed description of each variable.

However, the dot plot representation highlighted the range of actual values for each variable (**Figure 1**). The range was quite limited for a few items such as sexual behaviours (0.0–5.6%), allogrooming (0.0–11.1%), or the crouched body posture (0.0–6.7%). For most variables, however, the percentages of occurrence were much more widely distributed: inactivity (8.9–90.0%), SB (0.0–53.3%), seated (13.3–92.2%) or “on bars” (1.1–85.6%) body postures, revealing high inter-individual variations.

Multiple component analysis (MCA) was performed in order to analyse such great inter-individual variability (**Figure S1**). Short distances between variables and individuals mean that these individuals display high scores for these variables, i.e. alike individuals display similar behavioural profiles. The first factorial plane, defined by two dimensions (i.e. Factors 1 and 2), of the analysis accounted for 26.9% of the total variance (Factor 1 accounted for 13.9% and Factor 2 for 13.0%). On the first axis (Factor 1), the locomotion, “on bars” posture and upper location items were strongly opposed to maintenance, displacement behaviours, bottom location and seated posture items. On the second axis (Factor 2), behaviours directed toward the observer in a four-legged posture and therefore a body facing the ground were opposed to inactivity facing the wall in the upper part of the cage.

**Figure 2:**
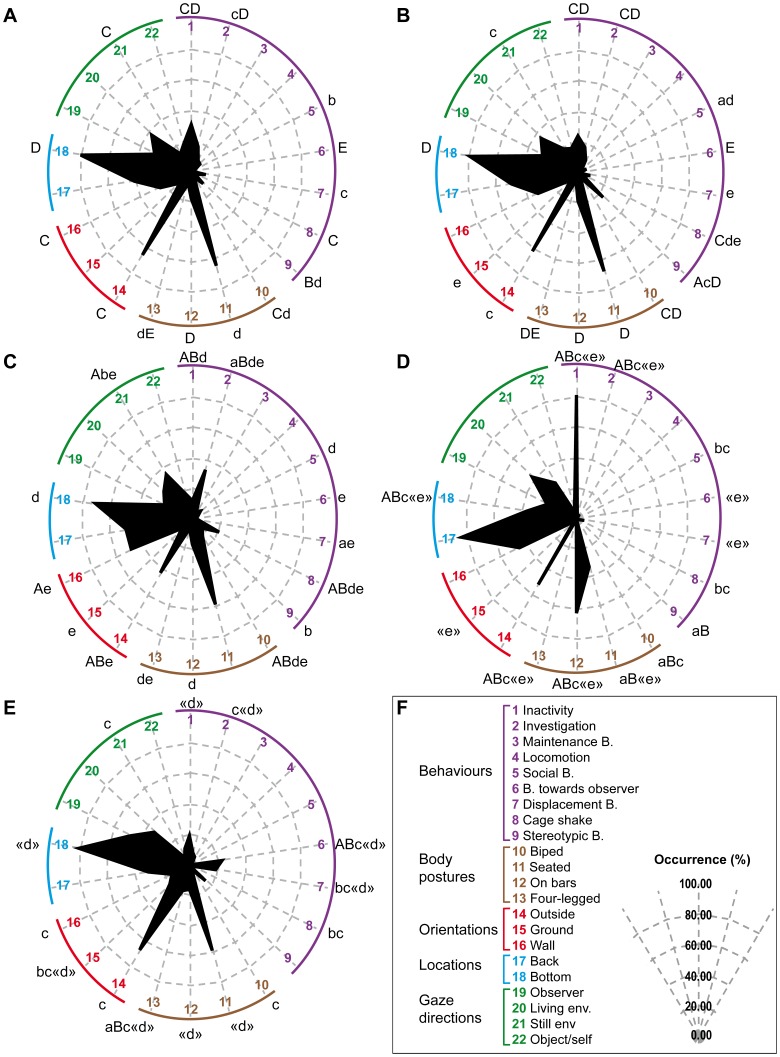
Five behavioural profiles resulting from hierarchical cluster analysis. Following the MCA (**Figure S1**) of the single-housed animals, a hierarchical cluster analysis was performed and resulted in 5 groups (n_A_ = 14, n_B_ = 14, n_C_ = 5, n_D_ = 4, n_E_ = 3). For each variable collected, the mean percentages of occurrence were calculated among the 5 groups. The radar profiles of group A (**A**), group B (**B**), group C (**C**), group D (**D**) and group E (**E**) were created using a selection of collected variables (**F**). The radar legend of the radars is explained on panel **F**. Each axis of the radar indicates the mean percentage of occurrence for a given variable: a behaviour (from 1 to 9), a body posture (from 10 to 13), a body orientation (from 14 to 16), a location in the cage (from 17 to 18) or a gaze direction (from 19 to 22). The abbreviations “B.” and “env.” stand for “behaviour” and “environment”. On graphs **A to E**, significant p-values in Mann-Whitney U tests before (small-letters, p<0.05) and after (capital letters, p<0.005) a Bonferroni adjustment are indicated. In front of each axis, the letters represent the groups versus which the p-values are significantly different for a given variable. P-values between quotation marks (« ») indicate significance (p<0.05) if small group size correction was not applied. See **Table 3** for detailed time budget per cluster and **Table S1** for full Kruskal-Wallis statistics.

Although the MCA provides invaluable information on the main behavioural characteristics of the individuals, it can only be represented on 2-dimension graphs (each axis being one factor) and, therefore, does not bring to light the inter-individual variability in its entirety. We, therefore, submitted our data to hierarchical cluster analysis in order to accurately identify the groups of individuals displaying similar profiles. This resulted in five distinct clusters, named A (n = 14), B (n = 14), C (n = 5), D (n = 4) and E (n = 3) groups (**Figure 2**). Salient results are displayed on radar graphs in **Figure 2** while comprehensive statistical analysis is presented in **Table 3** **(**see **Table S1** for full Kruskal-Wallis statistics).

**Table 3:** Behavioural profiles of the 5 clusters and statistical comparisons.

Variables (% ± SEM; p)	group A; n = 14		group B; n = 14		group C; n = 5		group D; n = 4		group E; n = 3	
**Behaviours:**										
inactivity***	31.5±3.7	CD	24.8±1.7	CD	11.8±1.2	ABd	82.5±2.8	ABc”e”	20.0±5.2	“d”
investigation/manipulation**	17.6±3.1	cD	17.9±2.5	CD	33.3±2.5	aBde	2.2±1.1	ABc”e”	8.9±1.3	C“d”
maintenance B.	9.2±2.0		10.3±1.7		5.6±1.6		3.9±1.9		7.0±3.7	
locomotion	8.2±2.8		3.6±0.7		7.8±1.8		0.8±0.5		3.0±0.4	
social B.* :	2.1±0.5	b	6.7±1.3	ad	4.4±1.1	d	0.8±0.5	bc	1.5±0.7	
allogrooming**	0.1±0.1	B	2.7±0.9	A“d”	0.4±0.4		0.0±0.0	“b”	0.0±0.0	
B. towards human* :	2.5±1.1	E	1.0±0.4	E	3.1±1.8	e	0.0±0.0	“e”	24.1±5.2	ABc“d”
threat*	1.4±0.7	E	0.7±0.3	E	1.6±1.5	e		“e”	23.7±5.15	ABc”d”
submission**	0.6±0.6	“c”	0.0±0.0	“c”	1.6±1.0	“a””b”			0.0±0.0	
displacement B.* :	10.6±1.5	c	9.0±1.4	e	3.8±1.7	ae	5.8±2.5	“e”	19.2±4.8	Bc“d”
vacuum chew	2.4±0.7		2.2±0.4		1.1±0.6		2.2±0.9		8.9±1.7	
yawn	2.2±0.4		3.2±1.1		1.3±0.9		1.7±1.0		4.4±2.2	
scratch*	5.9±1.3	c	3.6±0.8		1.3±0.4	a	1.9±1.2		5.9±2.6	
cage shake***	3.2±0.9	C	4.8±1.1	Cde	21.1±2.8	ABde	0.8±0.8	bc	0.4±0.4	bc
stereotypic B.*	11.3±3.8	Bd	24.2±2.9	AcD	9.6±4.5	b	2.2±1.2	aB	15.5±7.4	
feeding B.*	8.3±1.9	bd	2.7±0.7	ade	2.2±1.0	e	0.6±0.3	ab“e”	7.0±1.0	bc“d”
lipsmacking	8.5±3.3		9.4±3.5		18.2±8.2		21.1±10.2		11.5±4.9	
vocalization	7.9±2.7		8.6±2.0		11.3±3.7		6.1±4.7		5.2±3.0	
Behavioural switch**	64.5±2.5	cD	69.6±1.6	cD	76.4±1.4	abd	32.8±6.2	ABc“e”	66.3±3.5	“d”
Behavioural diversity***	16.2±0.7	BD	20.2±0.7	AD	19.0±1.0	d	8.0±1.3	ABc“e”	16.7±1.4	“d”
**Body postures:**										
biped**	4.1±1.2	Cd	3.3±0.4	CD	13.6±2.2	ABde	0.0±0.0	aBc	3.3±1.7	c
seated*	64.2±4.8	d	67.5±3.6	D	58.7±5.6		33.0±6.9	aB“e”	59.2±8.5	“d”
on bars*	17.3±3.6	D	18.2±3.2	D	16.9±6.6	d	62.5±8.6	ABc“e”	15.9±6.2	“d”
four-legged**	7.7±1.9	De	5.0±0.9	DE	6.9±1.0	de	0.3±0.3	ABc“e”	17.4±1.6	aBc“d”
slumped	5.9±2.1		4.4±1.1		2.2±1.0		4.2±2.9		3.0±1.0	
***Main B. while slumped:***	**n = 10**		**n = 13**		**n = 4**		**n = 2**			
inactivity	8.7±3.7		12.0±5.8		0.0±0.0		62.5±37.5		0.0±0.0	
investigation/manipulation	11.8±9.8		19.5±7.8		61.7±21.7		0.0±0.0		11.1±11.1	
maintenance B.	49.5±12.4		44.1±11.0		33.3±23.6		25.0±25.0		58.3±30.0	
**Body orientations:**										
outside *	64.7±2.1	C	61.2±5.2	c	42.2±3.0	Abe	51.7±16.7		67.0±7.5	c
ground*	10.9±2.9		6.9±1.3	e	9.8±1.7	e	2.5±2.5	“e”	18.9±1.1	bc“d”
wall*	24.4±2.7	C	31.9±5.3		48.0±3.1	Ae	45.8±17.5		14.1±8.0	c
***Main B. while oriented*** * “* ***wall”:***										
displacement B.*	7.4±1.7	“d”E	7.0±2.6	e	2.5±1.3	e	0.8±0.8	“a” “e”	26.4±3.9	Abc“d”
inactivity**	20.9±3.2	cDe	12.5±3.1	D	6.5±1.8	ad	88.8±4.1	ABc“e”	3.7±3.7	a“d”
investigation/manipulation**	28.3±5.6	D	20.3±4.3	cd	35.1±3.9	bde	2.0±1.6	Abc“e”	15.9±2.6	c“d”
cage shake**	10.5±3.6	C	11.4±3.6	C	36.0±3.7	Abde	3.3±3.3	c	0.0±0.0	c
**Cage locations:**										
back	41.3±7.2		48.9±7.6		48.0±7.0		84.7±11.7		30.4±16.0	
bottom*	80.1±4.1	D	79.4±3.2	D	70.0±5.6	d	36.9±8.5	ABc“e”	81.5±7.5	“d”
sides**	60.9±4.0	b	75.1±3.5	ae	74.6±4.3		83.9±12.1		38.1±14.1	b
**Gaze directions:**										
observer	28.8±3.9		28.2±4.0		21.8±4.9		23.6±7.2		47.8±4.6	
still env.* :	14.4±1.9	C	19.2±3.6	c	34.0±5.4	Abe	28.6±10.3		6.3±3.6	c
wall*	9.9±1.6	Ce	14.1±3.3	ce	28.7±5.5	Abe	18.0±7.3		3.0±1.0	abc
manipulable object*	9.8±3.2	d	6.0±1.5	d	12.0±3.6	de	0.3±0.3	abc“e”	4.1±0.4	c“d”

The mean percentages of occurrence (with regard to the 90 scans) and standard error means (SEM) per cluster are reported below for a selection of collected variables. The “behavioural diversity” is a mean number of distinct behaviours observed during the 90 scans. The “behavioural switch” between successive scans was calculated using a score for each scan: 0 if the behaviour was the same as in the previous scan, or 1 if it was different; the scores were added up within one session and transformed in a percentage with regard to the 15 scans of a session. The abbreviation “B.” stands for behaviour. Significant p-values after Kruskal-Wallis test are indicated by stars (*) in the left column (*: p<0.05; **: p<0.01; ***: p<0.001). Significant p-values in Mann-Whitney U tests before (small-letters, p<0.05) and after (capital letters, p<0.005) a Bonferroni adjustment are indicated on the right side of the SEM. The letters represent the groups versus which the p-values are significantly different for a given variable. P-values between quotation marks (« ») indicate significance (p<0.05) if small group size correction was not applied. Statistics concerning the “behaviours expressed whilst in a slumped posture” included only the 32 individuals that expressed this body posture at least once during the observations (H_(4,32)_). Group sizes including these individuals are indicated in the corresponding columns. See **Table S1** for full Kruskal-Wallis statistics.

Both groups A and B (**Figure 2**) showed high levels of cage investigation (A: 17.6±3.1% and B: 17.9±2.6%), maintenance activities (A: 9.2±2.0% and B: 10.0±1.7%) and displacement behaviours such as yawning, scratching and vacuous chewing (A: 10.6±1.5% and B: 9.0±1.4%). Group A individuals, however, ate 8.3% (±1.9%) of the time, and expressed 11.3% (±3.8%) of SB, while group B expressed 6.7% (±1.3%) of social behaviours, 24.2% (±2.9%) of SB and a higher behavioural diversity (20 behaviours vs. 16 in group A). Both groups were mainly located in the bottom side parts of the cage, similarly at the front or the back (bottom A: 80.1±4.1% and B: 79.4±3.2%; side A: 60.9±4.0% and B: 75.1±3.5%; front A: 58.7±7.2% and B: 51.1±7.6%), sitting most of the time (A: 64.2±4.8% and B: 67.5±3.6%) with a body oriented in majority toward the exterior of the cage (A: 64.7±2.1% and B: 61.2±5.2%) (**Table 3**).

Group C expressed the highest level of cage investigation (33.3±2.6%), cage shaking (21.1±2.8%) and the lowest level of inactivity (11.8±1.2%) (**Figure 2**). They switched from one behaviour to another between successive scans more often than their peers (76.4±1.4%). Similarly to groups A and B, they were mainly located in the bottom side parts of the cage (bottom 70.0±5.6%; side 74.7±4.3%; front 52.0±7.0%) in a seated posture (58.7±5.6%). They, however, faced the wall in 48.0% (±3.1%) of the scans and looked at the wall 28.7% (±5.5%) of the time. When facing the wall, they mainly investigated the environment (35.1±3.1% of time spent facing the wall) or shook the cage (36.0±3.8% of time spent facing the wall) (**Table 3**).

Group D was characterized by a high level of inactivity (82.5±2.8% of the time), and low levels of cage investigation (2.2±1.1%) and SB (2.2±1.2%) (**Figure 2**). These individuals displayed the lowest behavioural diversity (8 behaviours) and switched behaviours in only 32.8% (±6.2%) of successive scans. They were mainly located in the back upper corners of the cage, hanging on bars more than their peers (62.0±8.6% of the time). Similar to group C, they faced the wall as often as the exterior. Unlike group C, however, these individuals were inactive 88.8% (±4.1%) of the time spent facing the wall (**Table 3**). They were also inactive 62.5% of the time spent in the slumped body posture, while individuals from group A, B, C and E were mainly expressing maintenance behaviours or environment investigation (**Table 3**). The inactivity level whilst in a slumped posture tended toward statistical significance (H _(4, 32)_  = 9.026184; p  = .0605) despite the small number of individuals in this group (n = 4).

Group E animals frequently threatened the observer (23.7±5.1%), ate 7.0% of the time during the scans (±1.0%) and spent only 8.9% (±1.3) of the time in cage investigation (**Figure 2**). These animals were mainly located at the middle front bottom of the cage (bottom 81.5±7.5%; side 38.1±14.1%; front 69.6±16.0%). They stood on all four limbs in 17.4% (±1.6%) of the scans. Interestingly, this group expressed the highest level of displacement behaviours (19.2±4.8%). Although not significantly different from the other groups, these 3 monkeys stared at the observer in 47.8% (±1.3%) of the scans (**Table 3**).

**Figure 3:**
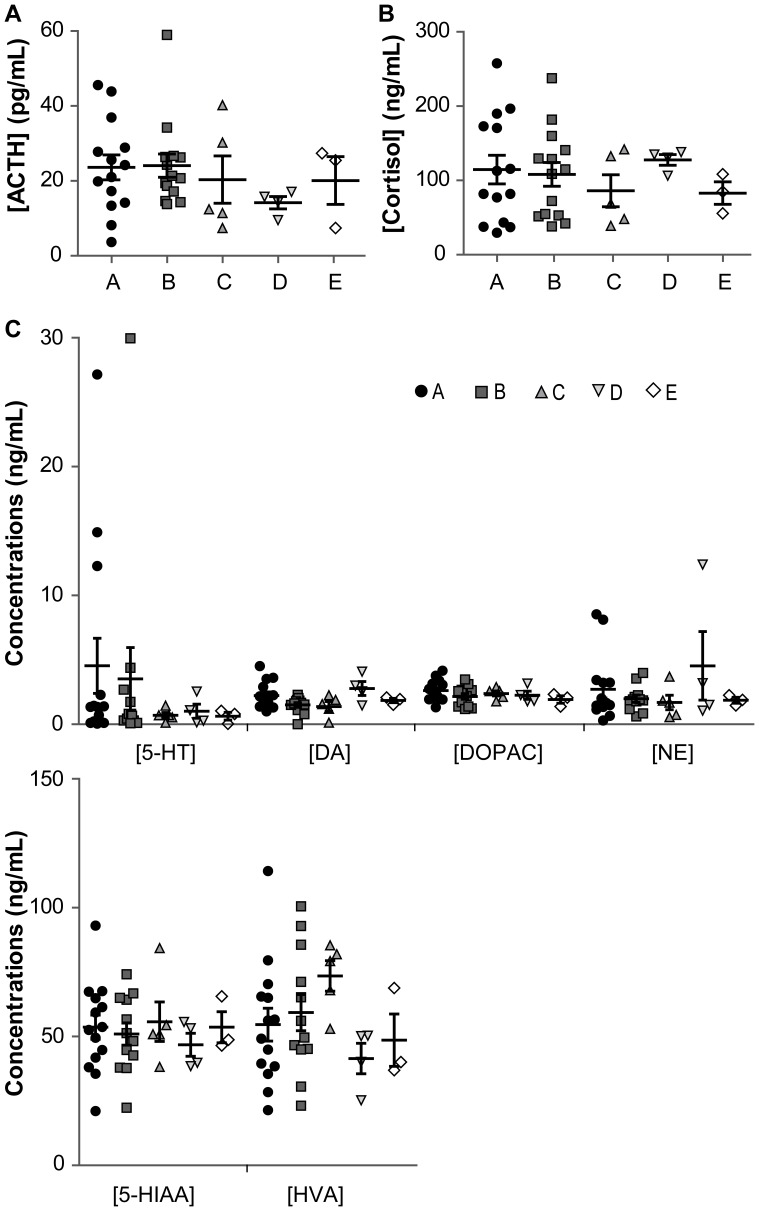
Physiological profiles of the 5 behaviourally-discriminated groups. The mean concentrations (± standard error means) of plasmatic ACTH (pg/mL) (**A**) and plasmatic cortisol (ng/mL) (**B**) and of CSF serotonin (5-HT), CSF 5-hydroxyindoleacetic acid (5-HIAA), CSF dopamine (DA), CSF homovanillic and CSF 3,4-Dihydroxyphenylacetic acids (HVA and DOPAC), and CSF norepinephrin (NE) (**C**) are presented for the 5 hierarchical cluster analysis-resulting groups (n_A_ = 14, n_B_ = 14 in plasma and 12 in CSF, n_C_ = 5, n_D_ = 4, n_E_ = 3) on panels **A, B** and **C**, respectively. Symbols indicate individual values from groups A (black circle), B (grey square), C (dark grey triangle pointing up), D (light grey triangle pointing down) and E (white diamond shape) while black lines indicate the group means. See **Table 4** for mean concentrations per cluster and **Table S1** for full Kruskal-Wallis statistics.

Most experimentally-induced animal models have so far been built around a neurobiological hypothesis. For instance, dysfunctions in both the monoaminergic systems and the hypothalamic-pituitary-adrenocortical (HPA) axis are thought to be related to several mental disorders, such as depressive or anxiety disorders [23], [46], [47]. Although the construct validity of the approach is undermined by the lack of actual monoaminergic or HPA-related biomarker of these diseases [1], [47]–[51], we here questioned whether the atypical behavioural profiles are associated with a particular physiological profile. We hence measured the adrenocorticotropic hormone (ACTH) and cortisol plasma levels as well as the cerebrospinal fluid (CSF) levels of three major monoamines, i.e. dopamine (DA), norepinephrine (NE) and serotonin (5-HT), and their metabolites. The mean concentrations were calculated for each of the 5 groups of animals that had been identified on the basis of behavioural discrimination and the results are shown in **Figure 3** and **Table 4** (see **Table S1** for full Kruskal-Wallis statistics). No significant difference was found across the groups for any marker (**Figure 3**), although some trends might deserve further investigation in a greater number of animals (e.g. a trend towards a decrease in ACTH in group D – **Figure 3A**- or an increase in HVA in group C – **Figure 3C**).

**Table 4:** Physiological profiles of the 5 behaviourally-discriminated clusters.

Variables (mean ± SEM)	group A; n = 14	group B; n = 14	group C; n = 5	group D; n = 4	group E; n = 3
**Plasmatic concentrations**					
[ACTH] (pg/mL)	23.6±3.3	24.1±3.1	20.3±6.3	14.2±1.6	20.1±6.4
[cortisol] (ng/mL)	114.6±19.2	108.2±16.0	86.1±21.5	127.5±7.2	83±15.2
**CSF concentrations (ng/mL)**		**n = 12**			
[5-HT]	4.5±2.1	3.5±2.4	0.7±0.2	1±0.5	0.6±0.3
[HIAA]	53.6±4.7	51±4.3	55.7±7.7	46.8±4.5	53.6±6.0
[DA]	2.2±0.3	1.5±0.2	1.4±0.4	2.8±0.5	1.8±0.2
[DOPAC]	2.6±0.2	2.2±0.2	2.4±0.2	2.3±0.3	1.9±0.3
[HVA]	54.6±6.4	59.3±7.0	73.5±5.9	41.5±5.9	48.6±10.2
[NE]	2.7±0.7	2±0.3	1.7±0.6	4.5±2.7	1.9±0.2

The mean plasmatic or spinal concentrations (± standard error means) per cluster are reported below for the following measured variables: adrenocorticotropic hormone (ACTH); cortisol; serotonin (5-HT); 5-hydroxyindoleacetic acid (5-HIAA); dopamine (DA); homovanillic acid (HVA); 3,4-Dihydroxyphenylacetic acid (DOPAC); and norepinephrine (NE). No statistical difference was found (see **Table S1** for full Kruskal-Wallis statistics).

## Discussion

In this preliminary study, we describe behavioural profiles, as well as some associated biochemical features, in forty three-year old male cynomolgus monkeys living in single cages in a farming environment. Five statistically-distinct behavioural profiles were identified using reliable unbiased multifactorial analyses and hierarchical clustering. We propose that animals from the low n groups (D and E) display behaviours that are reminiscent of psychiatric disorders such as depressive and generalized anxiety disorder respectively. Further analyses are needed to evaluate whether the three other profiles identified are more reminiscent of “typical” behavioural profiles or of other pathological syndromes. This study reveals that in the same environmental conditions, different individuals develop distinct behavioural syndromes.

In the wild, cynomolgus monkeys live in multi-male, multi-female groups with males emigrating when they reach sexual maturity. Females remain in their natal troops and exhibit strong matriarchal dominance hierarchies [22]. Weaning of the young occurs progressively with decreasing mother-infant body contact, infant suckling and increasing maternal rejection behaviours until nursing completely stops around 10 to 11 months of age [52], [53]. Chinese breeding farms commonly adopt a one-male, multi-female group housing for breeding purposes. Weaning takes place around the age of 6-months. Following this, animals are group-housed with peers of approximately the same age until the age of 3 years, after which males are housed singly, until being exported for research purposes in the US, Japan and Europe. Nevertheless, among the single-housed individuals, we observed several wild-like behaviours (present in the natural behavioural repertoire) such as locomotion, investigation, support shaking, maintenance activities and even social behaviours. Except for maintenance activities and inactivity, the frequency of these behaviours was decreased compared to wild animals [24]. This could be explained at least in part by the restricted living space, the absence of predators and congeners, and the availability of food at regular times associated with single housing. Unsurprisingly, macaques expressed stereotypic behaviours (SB) for 15.0% of the time on average. Captivity and single housing have previously been reported as risk factors for the development of such atypical behaviours in macaques [54]–[56] and it is well-known that stressful conditions are most likely to induce atypical behaviours in horses and pigs [11], [57], [58] or mental disorders in humans [2]. Although 97.5% of individuals expressed SB at least once, its occurrences ranged from 0 to 53.3% of the scans. Similarly wide distributions were observed for most behaviours. Such inter-individual differences have previously been described for atypical and typical behaviours in NHPs [59], [60] and support our choice to discriminate profiles using multifactorial correspondence and hierarchical cluster analyses.

In the MCA, the first factorial plane accounted for 26.9% of the total variance, which is relatively low but is likely explained by the large number of active modalities. We intentionally included as many modalities as possible so that no biased choice of the observed parameters could interfere in the analysis. With common statistical tests, we then assessed significant differences between the clusters establishing the relevance of the descriptive analyses. On the basis of scan-sampling observations submitted to factor and cluster analyses, we reported 5 distinct profiles, three of which were not reminiscent of known disorders while two could be reminiscent of symptoms of human mental disorders, such as depressive and generalized anxiety disorders.

Group A, B and C profiles suggested that they may have been less affected by their living conditions than groups D and E, attempting to reach their neighbours for social contact, investigating their cages and expressing behaviours relating to tension (e.g. support shaking [61]). The most “typical” animals were probably the group A animals, which showed mostly investigation, maintenance and locomotion. Nevertheless the animals of these three profiles, and especially group C, also expressed abnormal behaviours such as SB and many spent time facing the wall, showing that they still were sensitive to these impoverished conditions. Single housing has been reported as a risk factor for the development of atypical behaviours in macaques [56] and a retrospective study (including 362 subjects) indicated that 88.7% of captive single-housed macaques expressed at least one kind of SB [54]. Some authors have suggested that SBs are a way of coping with stressful conditions, thus avoiding more pathological syndromes by being “active” [14], [55], [62], [63]. Longitudinal studies would be needed in order to see whether these apparent differences might be due to timing (i.e. would they appear in all animals with time). The difference between the profiles of groups A, B and C could be explained by different temperament or “personality” traits, defined as an individual’s perception of its situation and behavioural response to it [64]. In the literature, the concept of “personality” has indeed been used in many species as an explanation for phenomena such as dominance status, differences in social behaviours, or reactivity towards challenging stimuli [60], [64]–[68]. It is presently unknown whether a relationship exists between “personality traits” and the emerging pathological profile in unfavourable conditions (i.e. could the group A, B and C monkeys have been more “extravert” than the D and E monkeys expressing their response to their environment by being inactive or producing displacement behaviours?).

More interesting with respect to the possible identification of NHP equivalents of neuropsychiatric conditions are the individuals from groups D and E. Group D monkeys clearly stood out with their high level of inactivity, the lowest behavioural diversity and behavioural switches, the rare expression of maintenance activities and the presence of a slumped body posture. These features could be reminiscent of 3 key DSM-IV criteria of major depressive disorder [10], namely: the decrease of interest in usual activities, the psychomotor slowdown, and energy loss. These characteristics were consistent with the ones of “depressed” macaques described in the literature (e.g. “slumped body posture, inactivity with open eyes, and a lack of responsiveness to environmental events” [29], [30]; decreased locomotion and self-grooming rates, “the appearance of curling up into a ball” [26], [37]), except for the slumped body posture, that was not the most frequent body posture observed here and was not specific to this group of individuals. The few occurrences of this posture could be explained by the smaller amount of time spent in single cage prior to the beginning of our study and the increased behavioural and physiological consequences of social isolation with time [25], [69]. The difference between the groups regarding the slumped body posture could be related to the type of behaviour expressed whilst in this posture. While individuals from group D were inactive 62.5% of the time spent slumped, individuals from group A, B, C and E were mainly expressing maintenance behaviours or environment investigation. These differences tend towards statistical significance. However the orientation towards the wall was clearly and statistically associated with inactivity in this profile rather than with displacement activities in profile E or with investigation and cage shaking in profile C. The orientation towards the wall could be relevant as a marker of depressed state when associated with inactivity. In addition, these animals D displayed a frequent location at the back of the cage, and few gazes directed to manipulable objects. These elements could be interpreted as withdrawal and loss of interest in the environment and point toward depressive-like symptoms as well. As depressive behaviours when single-housed are not necessarily predictive of such behaviours in social housing conditions (as reported in [29]: monkeys depressed in a social group did not display depressive-like behaviours in a previous phase when submitted to a one year-long single housing), one must remain cautious. Nevertheless, the present “depressive-like” monkeys presented characteristics similar to those of “withdrawn” horses (e.g. atypical posture, unusual gaze, and indifference to environmental stimuli) that have recently been proposed as an ethological model of depression [70].

While the four other groups differed on several levels, they were all active and expressed a considerable amount of SB compared to individuals from group D that remained mostly inactive and did not express any SB. Although these behaviours seem acknowledged as an indicator of poor well-being and suboptimal housing conditions [14], [55], their absence is not sufficient to qualify an environment as optimal to a species. This further suggests that group D profile rather reflects a “despair” behavioural state. Yet the association of SB with body postures and orientations could be used to identify typical and atypical profiles that might reflect distinct susceptibilities towards diverse future pathologies. Further investigations are thus required to assess the consistency of this depressive-like profile.

Individuals from group E had 2 specific attributes. On the one hand, they expressed the highest level of yawns, scratches and vacuous chewing. These behaviours are often called displacement activities because they are not associated with their usual causes (namely tiredness, itching and eating) and occur in situations where one would not expect to observe them [71], [72]. In stressful situations, humans and NHPs display such behaviours, which have been reported as modelling anxiety due to their dose-dependent increased and decreased frequencies after anxiogenic FG 7142 and anxiolytic lorazepam administration, respectively [73]. On the other hand, group E macaques displayed a high level of aggressiveness towards the observer (threat face and vocalizations) associated with stares, a quadrupedal body posture and a frequent location at the front of the cage. These are common adaptive responses to a staring human intruder from whom the monkey cannot flee [31], [74]. In the present study, however, the non-staring observer, though present in the room, watched the animals via a video-camera. Such indirect observation is less threatening [74], [75] and rarely elicited aggressive responses in the other individuals of our study. In the literature, monkeys usually displayed freezing or submissive behaviours rather than aggressiveness when confronted to a non-staring passive observer [74], [75]. Moreover animals were recorded during six distinct 30-minute sessions, with a total of 9 hours spent in each observation room, and should have habituated to the observer presence. Although cynomolgus monkeys habituate more slowly than rhesus macaques to human presence [75], groups A, B and C did not respond as vividly as group E (threats directed toward the observer). The presence of the displacement behaviours led us toward the definition of anxiety disorders in the DSM-IV [10]. Both specific behaviours identified in group E (displacement and aggressive behaviours) could recall 3 diagnostic criteria of generalized anxiety disorder: anxiety, restlessness, and irritability. The last 2 criteria can be associated with other diseases, such as depressive disorder or attention deficit and hyperactivity disorder (ADHD) [10]. Though completely different from the depressive-like group, it would be of interest to further characterize the anxiety-like features of these individuals.

We consider the lack of obvious dysregulation of either the plasma ACTH or cortisol levels, or of the CSF monoamines as supportive of our behavioural findings on the ground of lack of biomarkers for human psychiatric diseases [48], [49]. Although it is believed that dysfunctions in monoaminergic systems and hypothalamic-pituitary-adrenocortical (HPA) axis are related to several mental disorders, especially depressive and anxiety disorders, respectively [23], [46], [47], none of these hypotheses are fully acknowledged thereby opening a promising line of investigation. Indeed the literature contains numerous contradictory studies reporting either modifications or no change in these parameters. For instance, no significant difference in CSF tryptophan, 5-HIAA, or NE concentrations was reported between normal volunteers and depressive patients [76]. Decreased or unaltered plasma cortisol levels were described in chronic depressive patient in another study [51]. Such contradictory results were also reported in preclinical studies modelling depressive and anxiety disorders. Either decreased or increased levels of 5-HIAA and HVA in CSF were observed in monkeys whether they were reared among peers [77], [78] or with variable foraging demand-assigned mothers, a rearing condition used to study anxiety traits [79]. The most convincing demonstration of the dubious variation in these markers in depressive and anxiety disorders is the lack of blood tests for diagnosing them [48], [49] and the lack of such inclusion parameters in clinical trials, which usually require meeting diagnostic criteria for the disease of interest according to the DSM-IV and reaching a minimum score in the Hamilton Depression (or Anxiety) Rating scale. Confirmation of the lack of reliability of such “markers” is their total absence from 100 clinical trials held between 2002 and 2012 (Source: ClinicalTrials.gov), thereby indirectly questioning the construct validity of the experimentally-induced animal models. These considerations taken together with our results might suggest that physiological and neurochemical data alone are not sufficient to identify pathological susceptibilities among captive macaques and that behavioural features could be a more reliable first detection tool. Subsequent cognitive and emotional tasks must then be performed on these specific individuals for confirming (or not) the similarities with human disorders.

Three-year-old male long-tailed macaques are adolescents that have not reached sexual maturity [80]. To our knowledge, no study has exhaustively reported time budgets of long-tailed macaques according to gender and developmental stages. The expression of some behaviours are indeed known to change with puberty and the associated plasmatic testosterone increase starting between 2 and 3 years old in male rhesus macaques [81]. For instance, play decreases with puberty, while inactivity and sexual behaviours increase (rhesus: e.g. [81]; stumptail: e.g. [82]). In the 70′s, Southwick investigated time budgets of 10 subadult and 7 adult socially–housed male rhesus macaques and reported that the four predominant activities in subadults were: feeding (31.0%), investigation (24.7%), grooming (18.9%), and resting (14.4%), compared to: grooming (34.2%), resting (30.1%), feeding (15.9%) and investigation (15.7%) in adults [83]. However, these changes are not as drastic as the ones reported in our depressive-like individuals (e.g. 82.5% of time spent inactive and 2.2% of investigation). Moreover, the full set of studies conducted by Harlow and then by Suomi reported that “monkeys of all ages are capable of developing behavioral syndromes analogous to certain forms of human depression” [26]. Adolescent rhesus macaques submitted to social separations exhibited depressive-like reactions but only if they had undergone separations early in life. Also Coplan et al. have extensively investigated the biochemical and behavioural short- and long-term effects of a rearing with a mother submitted to variable foraging demand [79], [84], [85]. In Humans, depressive disorders have been reported as well in adolescent and children (namely early-onset depression, for review see [86]), with the same resistance-to-treatment rate as in adults [87]. The early weaning of our subjects might have been perceived by some of them as an early adverse event. Therefore the expression of depressive-like states after single housing among our 3 year-old males was not surprising and the characteristics of such profiles were unlikely due to age, although our subjects had not reached adult maturity at data collection time.

As in every experimental set up, our study has limitations. Firstly, we observed exclusively male NHPs, although mental disorders (especially depressive disorders) affect more women than men in the human population [10], [29]. Only male monkeys are singly-housed in Chinese farms; females are housed in social groups. Although no behavioural difference was reported between single-housed cynomolgus males and females [24], we have conducted another study among socially-housed females in similar farms using the same observational procedures to completely preclude a potential gender effect and to investigate more naturalistic housing conditions for this gregarious species [88]. We indeed found a similar depressive-like profile among socially-housed females, but no anxious-like profile as females expressed nearly no behaviour directed to the observer. Secondly, we have no information about the genetic background of the animals. Given the substantial contribution of genetic factors to most psychiatric disorders and the heritability of several behavioural and neuroendocrine inter-individual differences [1], [35], [62], [89], investigating the genetic similarities between the individuals of a same profile would be interesting. Finally, an important element in the diagnosis of mental disorders is the duration of the symptom manifestation: e.g. 2 weeks for a major depressive episode [10]. Future studies should take account of this important feature and involve re-observation at intervals.

### Conclusions

Inter-individual differences were observed in spontaneous behaviours through unbiased ethological observations among macaques living in common breeding farm conditions. No specific behavioural testing was required to discriminate between 5 behavioural profiles, 2 of which were reminiscent of certain depressive-like and anxiety-like symptoms. Our results suggest that NHPs (as humans and other species) differ in their ways of coping with stress. As humans, some individuals seem to be more severely affected by stressful events than others. The use of unbiased behavioural observations might thus allow the identification of animals representing models of human mental disorders and their most appropriate control groups.

## Supporting Information

Figure S1 **First factorial plane of the multiple component analysis.** Behaviours, postures, body orientations and locations expressed by the 40 single-housed cynomolgus monkeys were submitted to MCA. The individuals are represented by bold black letters, accounting for the clusters to which they belong according to the cluster analysis following the MCA. Squares represent active modalities: grouped behaviours, grouped postures, body orientations and locations in the cage. Big black squares contribute strongly to the variance of the sample. On each axis is reported the percentage of the total variance accounted for by each factor. The abbreviations “behav”, “L” and “O” stand for “behaviour”, “location” and “orientation”. See **Tables 1** **and** **2** for a detailed description of each variable.
(TIF)

Table S1 **Kruskal-Wallis tests comparing the 5 clusters for a selection of collected variables and corresponding p-values, related to** **Figures 2** **and** **3** **.** Bold statistics and p-values are significant (p<0.05). Statistics concerning the “behaviours expressed whilst in a slumped posture” included only the 32 individuals that expressed this body posture at least once during the observations (H_(4,32)_). As 2 CSF samples from group B were contaminated, the analyses of monoamines and their metabolites included 38 individuals (H_(4, 38)_) while the rest of the behavioural and physiological analyses included 40 animals. The following abbreviations are used in the table: KW: Kruskal-Wallis; B.: behaviour; env.: environment; ACTH: adrenocorticotropic hormone; 5-HT: serotonin; 5-HIAA: 5-hydroxyindoleacetic acid; DA: dopamine; HVA: homovanillic acid; DOPAC: 3,4-Dihydroxyphenylacetic acid; and NE: norepinephrine.
(DOCX)
