# Supplementary figures and images for: Behavioural Profiles in Captive-Bred Cynomolgus Macaques: Towards Monkey Models of Mental Disorders?
Source: PLoS One. 2013 Apr 29;8(4):e62141. doi: 10.1371/journal.pone.0062141 (PMC3639229; doi:10.1371/journal.pone.0062141)

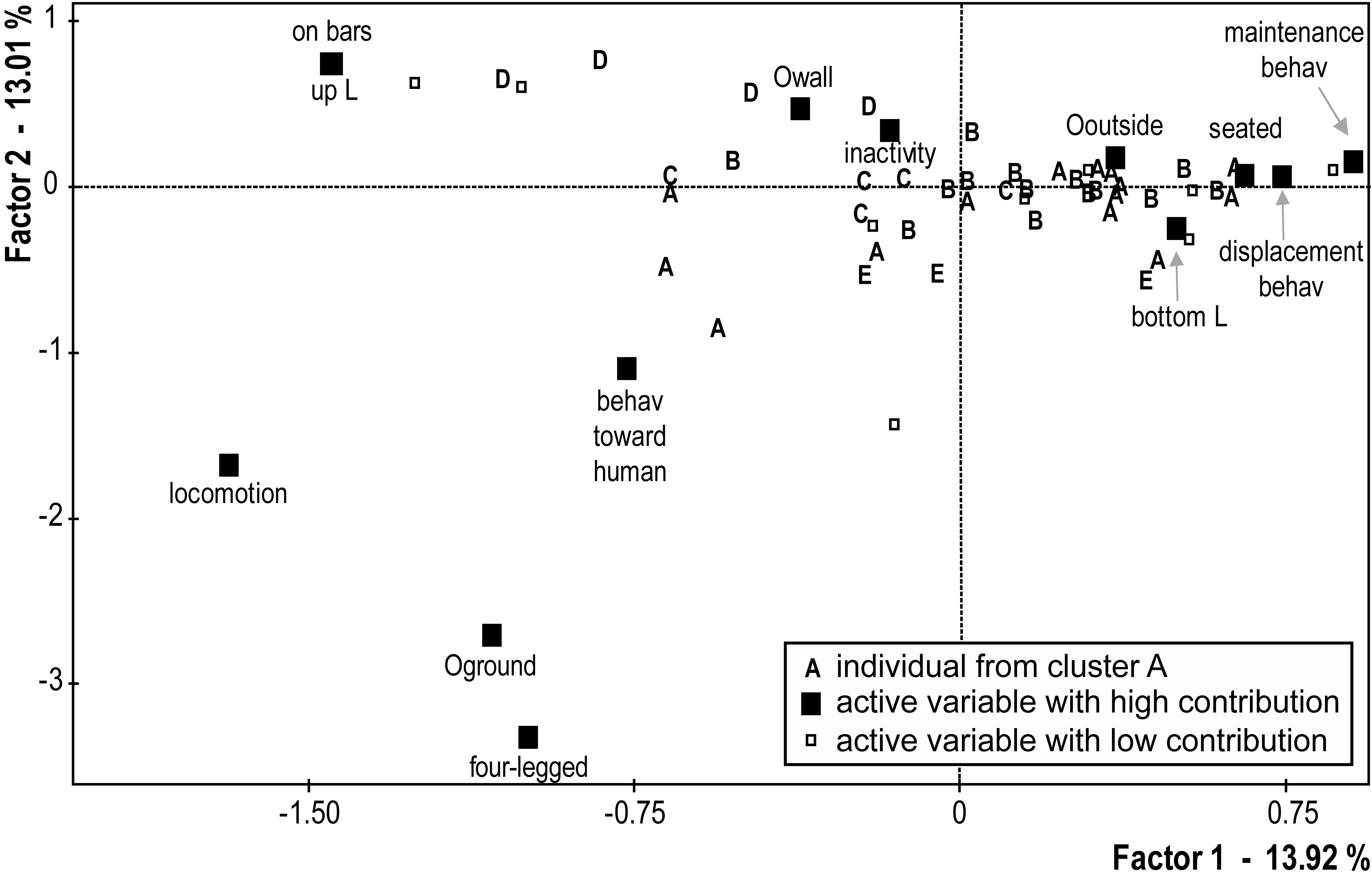

Supplement: Figure S1 — First factorial plane of the multiple component analysis. Behaviours, postures, body orientations and locations expressed by the 40 single-housed cynomolgus monkeys were submitted to MCA. The individuals are represented by bold black letters, accounting for the clusters to which they belong according to the cluster analysis following the MCA. Squares represent active modalities: grouped behaviours, grouped postures, body orientations and locations in the cage. Big black squares contribute strongly to the variance of the sample. On each axis is reported the percentage of the total variance accounted for by each factor. The abbreviations “behav”, “L” and “O” stand for “behaviour”, “location” and “orientation”. See Tables 1 and 2 for a detailed description of each variable. (TIF) [file pone.0062141.s001.tif]
