# Supplementary material for: Behavioural Profiles in Captive-Bred Cynomolgus Macaques: Towards Monkey Models of Mental Disorders?
Source: PLoS One. 2013 Apr 29;8(4):e62141. doi: 10.1371/journal.pone.0062141 (PMC3639229; doi:10.1371/journal.pone.0062141)
Supplement: Table S1 — Kruskal-Wallis tests comparing the 5 clusters for a selection of collected variables and corresponding p-values, related to Figures 2 and 3 . Bold statistics and p-values are significant (p<0.05). Statistics concerning the “behaviours expressed whilst in a slumped posture” included only the 32 individuals that expressed this body posture at least once during the observations (H(4,32)). As 2 CSF samples from group B were contaminated, the analyses of monoamines and their metabolites included 38 individuals (H(4, 38)) while the rest of the behavioural and physiological analyses included 40 animals. The following abbreviations are used in the table: KW: Kruskal-Wallis; B.: behaviour; env.: environment; ACTH: adrenocorticotropic hormone; 5-HT: serotonin; 5-HIAA: 5-hydroxyindoleacetic acid; DA: dopamine; HVA: homovanillic acid; DOPAC: 3,4-Dihydroxyphenylacetic acid; and NE: norepinephrine. (DOCX) [file pone.0062141.s002.docx]

| **Variables** | **Kruskall-Wallis H_(4,40)_** | **KW p-values** |
| --- | --- | --- |
| **Behaviours:** |  |  |
| inactivity | **20.74** | **0.000** |
| investigation/manipulation | **18.14** | **0.001** |
| maintenance B. | 4.80 | 0.309 |
| locomotion | 8.37 | 0.079 |
| social B. : | **13.02** | **0.011** |
| allogrooming | **15.79** | **0.003** |
| B. towards human : | **12.80** | **0.123** |
| threat | **13.01** | **0.011** |
| submission | **15.05** | **0.005** |
| displacement B. : | **11.69** | **0.020** |
| vacuum chew | 9.34 | 0.053 |
| yawn | 2.54 | 0.638 |
| scratch | **9.86** | **0.043** |
| cage shake | **19.29** | **0.000** |
| stereotypic B. | **16.71** | **0.002** |
| feeding B. | **11.67** | **0.020** |
| lipsmacking | 4.51 | 0.341 |
| vocalization | 3.18 | 0.529 |
|  |  |  |
| **Behavioural switch** | 17.72 | 0.001 |
| **Behavioural diversity** | 20.61 | 0.000 |
|  |  |  |
| **Body postures:** |  |  |
| biped | **18.24** | **0.001** |
| seated | **9.79** | **0.044** |
| on bars | **10.90** | **0.028** |
| four-legged | **15.72** | **0.003** |
| slumped | 1.16 | 0.885 |
| ***Main B. while slumped:*** |  |  |
| inactivity | 9.03 ^(4,32)^ | 0.060 |
| investigation/manipulation | 5.45 ^(4,32)^ | 0.244 |
| maintenance B. | 1.05 ^(4,32)^ | 0.902 |
|  |  |  |
| **Body orientations:** |  |  |
| outside | **9.75** | **0.045** |
| ground | **9.84** | **0.043** |
| wall | **10.51** | **0.033** |
| ***Main B. while oriented "wall":*** |  |  |
| maintenance B. | **12.63** | **0.013** |
| inactivity | **17.99** | **0.001** |
| investigation/manipulation | **13.45** | **0.009** |
| cage shake | **15.02** | **0.005** |
|  |  |  |
| **Cage locations:** |  |  |
| back | 7.76 | 0.101 |
| bottom | **12.54** | **0.014** |
| sides | **13.34** | **0.010** |
|  |  |  |
| **Gaze directions:** |  |  |
| observer | 6.59 | 0.160 |
| still env. : | **11.97** | **0.017** |
| wall | **12.30** | **0.015** |
| manipulable object | **11.79** | **0.019** |
|  |  |  |
| **Physiological samples:** |  |  |
| ACTH | 3.61 | 0.461 |
| Cortisol | 1.70 | 0.791 |
| 5-HT | 1.10 ^(4,38)^ | 0.894 |
| 5-HIAA | 0.66 ^(4,38)^ | 0.956 |
| DA | 5.57 ^(4,38)^ | 0.233 |
| DOPAC | 2.66 ^(4,38)^ | 0.616 |
| HVA | 6.10 ^(4,38)^ | 0.192 |
| NE | 0.93 ^(4,38)^ | 0.920 |
